# Preliminary Evaluation of a System with On-Body and Aerial Sensors for Monitoring Working Dogs

**DOI:** 10.3390/s22197631

**Published:** 2022-10-08

**Authors:** Marc Foster, Tianfu Wu, David L. Roberts, Alper Bozkurt

**Affiliations:** 1 Department of Electrical and Computer Engineering, NC State University, Raleigh, NC 27695, USA; 2 Department of Computer Science, NC State University, Raleigh, NC 27695, USA

**Keywords:** ECG, heart rate variability, electrodes, machine learning, 3D printing, wearable, drone, UAV, embedded system

## Abstract

This paper presents a system for behavioral, environmental, and physiological monitoring of working dogs using on-body and aerial sensors. The proof of concept study presented here includes two trained dogs performing nine scent detection tasks in an uncontrolled environment encompassing approximately two acres. The dogs were outfitted with a custom designed wearable harness to monitor their heart rate, activity levels and skin temperature. We utilized a commercially available micro-air vehicle to perform aerial sensing by tracking the terrain and movement of the dog in the outdoor space. The dogs were free to explore the space working at maximal speeds to complete a scent-based search-and-retrieval task. Throughout the experiment, the harness data was transferred to a base station via Wi-Fi in real-time. In this work, we also focused on testing the performance of a custom 3D electrode with application specific ergonomic improvements and adaptive filter processing techniques to recover as much electrocardiography data as possible during high intensity motion activity. We were able to recover and use 84% of the collected data where we observed a trend of heart rate generally increasing immediately after successful target localization. For tracking the dogs in the aerial video footage, we applied a state-of-the-art deep learning algorithm designed for online object tracking. Both qualitative and quantitative tracking results are very promising. This study presents an initial effort towards deployment of on-body and aerial sensors to monitor the working dogs and their environments during scent detection and search and rescue tasks in order to ensure their welfare, enable novel dog-machine interfaces, and allow for higher success rate of remote and automated task performance.

## 1. Introduction

Dogs play a critical role in modern society and perform an array of functions from simple quality of life interactions such as companionship, to professionally trained operations such as herding livestock; detecting drugs, explosives, and pests; and aiding in search and rescue (SAR) applications. A number of studies in the literature show that monitoring physiological signals (e.g., heart rate, heart rate variability) and behavioral indicators (e.g., activity levels, postures and body movement) not only provide insight into the physical and emotional health and welfare of the dogs, but also allow for computer-assisted training approaches [1,2,3,4,5,6,7]. Enabling such new information channels and decision-making capabilities during human and dog interactions through quantitative monitoring of dogs also allow for dog-machine or dog-robot interfaces [8,9,10,11,12,13].

Commercial unmanned air vehicle (UAV)-based surveillance has great utility in SAR applications for recording the search activity and surveying the environment. This is especially useful in relatively harsh environments that are challenging for human exploration but dogs can work relatively easily in, such as rubble piles after natural disasters or large agriculture fields and forests. In these environments, dogs can be excellent trackers on the ground where UAV cameras in the air allow for situational awareness and further insight into the detection tasks.

**Figure 1:**
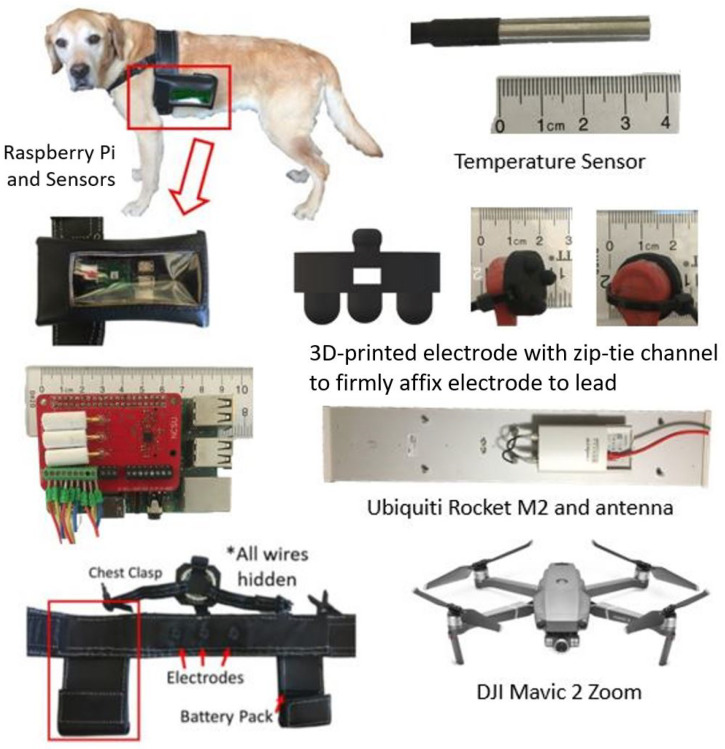
Hardware assembled to enable the on-body and aerial sensor system. A custom designed leather harness houses the microprocessor (highlighted by red box) and sensor in its pouches and hides all the wiring (bottom left). It also hosts custom 3D printed electrodes and temperature sensors. A commercial Wi-Fi router and antenna provide wireless connection to the Cloud. The aerial images acquired by commercial drone’s camera.

**Figure 2:**
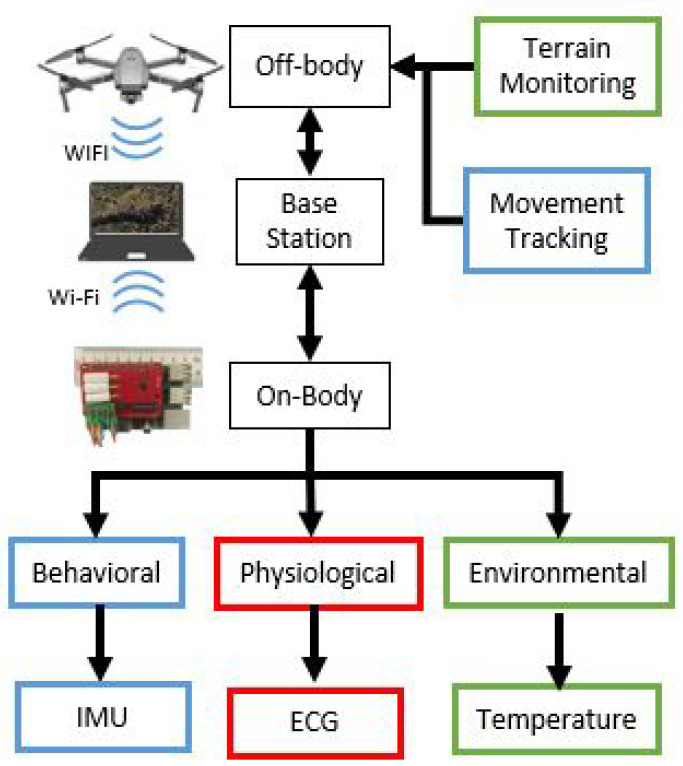
The block diagram describing the multimodal and distributed sensing capability of the presented on-body and aerial sensor system.

In this paper, we present our efforts towards combining the visual, environmental, and navigational sensors carried by a commercial UAV with a custom designed sensor-equipped canine harness [14,15,16]. We added features to our custom-equipped harness to support the ergonomic needs of the study; established a wireless network synchronously connecting all the sensors and actors together; and enabled a human-computer interface towards a dog-human-drone triad system as shown in Figure 1. We particularly focus on the assessment of critical information related to working dogs in the field using both harness based on-body and UAV based aerial data streams. Figure 2 displays an overview of the system. These include heart rate, heart rate variability, skin versus ambient temperature and micro- & macro-motion of the animal. The custom designed sensor system embedded into the dog harness monitors micro-environment—the immediate area of the dog. This system acquires local ambient temperature and high resolution body movement of the dog as it moves through the searched space. These are paired with the detection of key physiological signals of skin temperature, heart rate and heart rate variability. The UAV is used to track the gross movement in the macro-environment, or the larger working area the dog and handler conduct their activities in. This paper focuses on the preliminary assessment and analysis of the data streamed from both the systems with an emphasis on the development of signal and vision processing techniques related to the collected multimodal information.

The system described in this paper affords the first attempt towards fusing on-body and aerial sensors for multimodal interrogation of working dogs’ performance and wellbeing through data analytics that incorporate streams of physiological, behavioral and environmental data. We describe preliminary steps taken towards this goal and provide experimental results for proof of concept validation of sensing and system performance in a realistic wilderness environment. Analytical interpretation of sensor data reveals promising results, demonstrating that the sensing systems can work effectively in challenging environments and during high-level activity of the dogs.

In our prior work, we examined the benefits and limitations of various on- and off-body sensing of behavioral, environmental and physiological parameters for working dog applications [9,11,14,15,17,18,19,20,21,22,23]. The major challenge is the difficulty of monitoring dogs’ maximum heart rate during intense exercise due to excessive motion artifacts. We have made several design efforts to provide the best skin-electrode contact possible as discussed in Section 2.1 Materials and Methods: On-body Dog Harness System. We have also developed peak detection algorithm that can support the faster beat rate of a dogs heart during intense exercise (around 300 beats per minute) [24].

There has been a recent interest in enabling dog-drone or -ground robot interactions, especially for SAR applications. Previous research shows that dogs can be trained to follow a UAV or receive commands in such scenarios and engage in functional referential communication [25,26,27,28]. Previously, our team participated in the *Smart America Challenge* in 2013, organized by the White House in which a *Smart Emergency Response System* (SERS) was developed to connect cyber-physical technologies (robots and drones) with humans and instrumented dogs to help save lives in disaster areas [10,11]. In this, we developed a canine harness with environmental sensors to monitor the disaster area with cameras and gas detectors, as well as vibration motors and micro-speakers to communicate sound and tactile signals to the SAR dog. Drones established a Wi-Fi network and provided an overhead view of the canine and disaster area. Command/control center software developed by MathWorks, Inc. (Natick, MA, USA) connected dogs, drones, and other ground robots [29,30].

Another major effort to combine UAVs and working canines was a notable computer-networking-focused project, based in Europe, *Synergistic Interactions in Swarms of Heterogeneous Agents* (SWARMIX) [31]. The project focused on a SAR application, using “swinglets” to automatically scan an area using computer vision where the software also tracks human-canine teams using drones and communicates areas of interest to the human handlers. The SWARMIX approach used only single modality sensing from the UAVs and did not incorporate on-body physiological and behavioral sensing for the dogs.

## 2. Materials and Methods

### 2.1. On-Body Dog Harness System

The details of our harness system can be found in [15]. This system is based on Raspberry Pi3B (Raspberry Pi Foundation, UK) microcontroller with an attached custom circuit cape we developed for collecting electrocardiography (ECG) and inertial measurement (acceleration and gyroscopic motion) via a $I^{2}C$ communication bus. This custom circuit collects ECG data via AD8232 (Analog Devices, Inc. Wilmington, MA, USA) and inertial data via LSM9DS0 (STMicroelectronics N.V., Geneva, Switzerland) at sampling rates of 440 and 220 Hz respectively. A custom leather harness is used fitting the exact size of electronic boards and enclosures. For ECG we use custom designed 3D printed conductive electrodes placed ventrally across the chest of the dog [15]. For this study, we modified the electrodes to support high intensity activity by 3D printing a channel to zip tie the electrodes into the harness. The three-prong structure provides optimum stability at the point of contact and by firmly affixing the electrodes to the housing, we mitigated potential issues during high motion and prevented the electrodes from rolling and losing body contact. With this custom electrode, we do not need to shave the dogs and were able to maintain adequate skin contact throughout the experiment.

An 85 g 5000 mAh battery powered the system and this can be easily extended as dogs can carry much larger payloads on the order of several kilograms. In practice, we achieved up to 12 h of continuous data transmission of the system with the given battery. The twelve hours of transmission time far exceeds the working time an individual dog would be required to perform a scent detection task. In search and rescue scenarios, handler-dog teams take regular breaks throughout a deployment. While deployments may last days or weeks and teams may work 8–12 h per day, seldom would any team work more than a two hours without a substantial rest period. At this point, the dog would be given a break, the equipment would be removed and a fresh battery would be used for the dog’s next activity.

For this study, we distributed a set of temperature sensors DSB18B20 (Maxim Integrated, San Jose, CA, USA) across the harness to collect skin versus ambient temperature as a vital sign. Three waterproof DS18B20 temperature sensors were added onto the board via terminal blocks and digital inputs with a sampling rate of 0.5 Hz since temperature is expected to change slowly. One sensor was placed between the strap of the harness and against the chest of the dog to monitor skin temperature. A second temperature sensor was placed inside the electronic pouch environment which houses the electronics. Finally, a temperature sensor was placed to protrude out from under the harness to measure the ambient temperature in the environment. This allowed for a complete picture of the micro-environment of the dog during search activities. In addition, the temperature of the Raspberry Pi central processing unit (CPU) was monitored as previously we had seen performance issues when the CPU temperature increased too high, when the dog is working under sun or hot environments.

While it is possible to perform some amount of streaming data analysis on a Raspberry Pi microprocessor, doing so limits the ability to synchronize analysis with other streams of data collected from other sources (i.e., the drone, other wearables on other canines, etc.). Thus, for this article our approach was to leverage the Raspberry Pi primarily as the microcontroller to collect and transmit raw data to a central location where it could be synchronized with other sources.

As such, A modified version of the “CanineEvaluation” software [3] served as a base station and graphical user interface for data collection to communicate with the microcontroller on the harness and aggregate the data. The software affords an on-screen display showing the real time data transmission of ECG, inertial measurement unit (IMU) and temperature sensor from a Raspberry Pi 3B over Wi-Fi. In addition, annotations can be made during data collection.

### 2.2. Aerial Drone Sensors and Wireless Networking

A commercially available DJI Mavic II Zoom (SZ DJI Technology Co., Ltd., Shenzen, China) was flown by an experienced pilot and used to film and track the dog throughout the experiment. This drone has a 4K resolution camera and is capable of speeds up to 72 km/h and flight time of over 30 min. We synchronized the data streams of the drone video with the ECG, IMU, and temperature sensors by manual time markers applied at the beginning and end of the experiments. The experiments took place in an area of approximately two acres. We used a Rocket M2 2.4 GHz CPE AirMax router and AM-2G15-120 AirMax Sector 15 dBi 120 deg antenna (both from Ubiquiti, Inc., New York City, NY, USA). The hardware is specifically designed for harsh weather and outdoor environments, which makes it ideal for this type of experimentation. Prior to experimentation, we walked the boundary of the search area by venturing more than 150 m from the router without line of sight due to tree obstructions and did not lose signal. This allowed us to transmit the ECG and IMU data back to the base station in real time throughout the search area.

### 2.3. Experimental Protocol with the Dogs

All animal procedures were approved by the NC State University Institutional Animal Care and Use Committee (IACUC). We ran four experiments with two male Labrador Retrievers (four and eight years old). We had nine search-and-retrievals in an area of approximately two acres. We outfitted the dogs with the harness and hid typical toys, called “bumpers”, used in retrieval training. We used positive reinforcement operant conditioning to train the dogs to search the bumpers. The field was set up to allow the dog to search for bumpers safely. The handler walked and occasionally pointed the dog during the search, and retrievals were noted in the software. The two dogs had previously been acclimated to performing tasks while under drone surveillance and not affected by its operation.

### 2.4. Data and Image Processing

Three axes of the IMU provided an activity level vector. The vector was used to detect excessive motion and reduce motion artifact in the ECG signal. The activity level was averaged over 5-s windows. The activity and heart rate were examined 10 s before and after retrieval. In addition, the activity vector was used to perform adaptive noise cancelling in areas of excessive motion to provide more accurate heart rate estimation. The ECG system and our peak detection algorithm was previously validated against commercial devices for resting and average intensity level activities such as walking at a lower speed and regular pace [32]. Our efforts for improving the ergonomics to support higher walking- and running-speeds and use of adaptive signal processing helped us to recover majority of the ECG signal.

**Figure 3:**
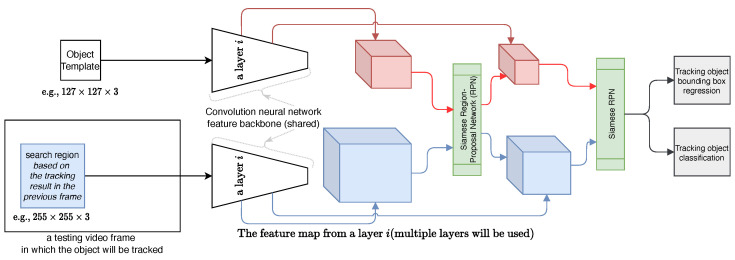
Illustration of the SiamRPN++ object tracking method, as edited from [33]. In tracking, given a target template (left-top) and search region (left-bottom), the network ouputs a dense prediction by fusion the outputs from multiple Siamese Region Proposal Networks (SiamRPNs). Each SiamRPN block is shown on right.

To demonstrate the functionality of the aerial camera sensing, we applied a state-of-the-art online single-object deep tracker, the SiamRPN method [33], to track the dog in videos. We first briefly summarize the workflow as illustrated in Figure 3, and then present the evaluation metric.

This online tracking algorithm has the following three components:

(i) Initializing the bounding box of an object instance to be tracked in a given frame of a video: Based on the initialization, a target model is learned (see Figure 3 top left diagram). With the recent resurgence of deep neural networks (DNNs), the target model is typically realized by a convolution neural network for better performance. In this paper, due to the movement of the drone while the camera records the videos, we apply multiple-frame initialization by manually selecting four to six frames (e.g., to account for the dogs disappearing and reappearing when they either are completely occluded by trees or bushes or run out of the camera field of view). The development of a fully automatic tracking algorithm is considered as a future work.
(ii) Tracking the object in the subsequent frames: For improved efficiency, a local search region is defined in the current frame based on the tracking result in the immediately previous frame (see Figure 3 left-bottom diagram). Then, the learned target model is used to “scan” the search region to detect the tracking object based on the scoring function determined by the nature of the target model.
(iii) Updating the target model based on the new tracking results: Since the initial target model is learned with a single observation of the object of interest in tracking, it is desirable to update the target model with more observations obtained to account for structural and appearance variations of the object of interest. Thanks to the expressive power of DNNs, the SiamRPN method enables us to obtain reasonably good tracking performance on the challenging drone videos.

In evaluating tracking performance, we annotated dogs in each frame of two videos with bounding boxes using the free and open source “labelImg” tool [34]. For a given video, we have the annotated ground-truth bounding boxes and the predicted bounding boxes by the tracking algorithm. We adopt the widely used single object tracking evaluation protocol which measures the success rate, precision and *normalized precision* between the tracking results and the annotated dog bounding boxes, which are summarized as follows.

To characterize *the success rate of tracking*, the intersection over union (IoU) between the ground-truth box and the prediction box is calculated in each frame, and then the success plot is generated by varying the IoU thresholds from 0.05 to 1.0 (inclusive) with a step size 0.05. For a given IoU threshold, the tracking is said to have succeeded in a frame if the IoU between the ground-truth box and the tracking result is greater than or equal to the given threshold. The success rate for a given threshold is the proportion of frames with successful tracking results. The mean success rate is the average over all the IoU thresholds.

To characterize *the precision* of tracking, the distance between the center of the ground-truth box and the center of the prediction box is calculated. In our case, the distance threshold varies between 0 and 51 pixels with a step size of 1. For a given threshold, *the precision rate* is defined by the proportion of frames with the center distances smaller than or equal to the threshold. The *mean precision rate* is the average over all thresholds. *The normalized precision* of tracking is calculated using both the center of ground-truth boxes and the center of prediction boxes, and normalizing those values by dividing each according to the side length of ground-truth boxes.

Intuitively, *the precision rate* only evaluate the relative distance between the ground-truth and the prediction without checking the shape and size of the boxes. The success rate captures both the distance and the shape and size.

Video files from the UAV camera were archived on local storage to a central repository in the cloud before backup for subsequent analysis during training of the SiamRPN++ model. It should be noted that, although not implemented here, the SiamRPN++ model, when doing inference as opposed to training, can run on a suitably equipped mobile device (e.g., a gaming laptop with discrete GPU or a single board computer miniaturized computer). However, while training, the requisite compute resources are more significant and thus the workload is better performed in the cloud.

## 3. Results

We set out to measure behavioral, physiological, and environmental data using both on-body harness and off-body UAV based aerial sensing in dogs during scent detection tasks.

**Figure 4:**
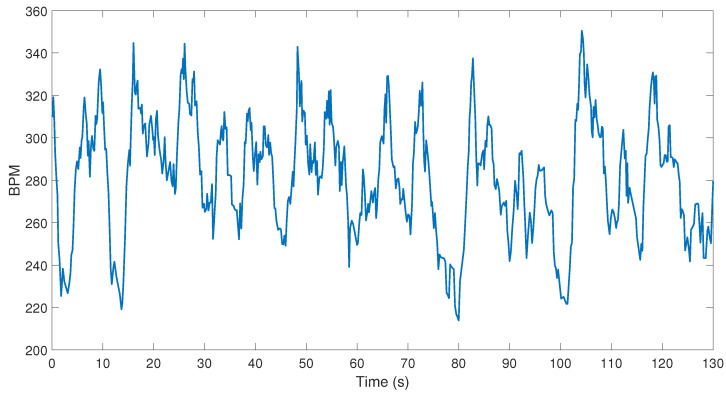
Initial heart rate estimation without adaptive filtering.

We used ECG waveforms to extract heart rate based on R peak detection. Initially, we extracted heart rate from the ECG signal using the “find peaks” function in MATLAB where peaks are detected with a minimum local maximum of three times the average signal value and minimum gap of 143 ms per peak. The initial analysis showed exceptionally high heart rates during intense activity above the expected maximum, as a result of motion artifacts. The observed instances of heart rate in the order of 360 beats per minute (bpm) is well above the expected physiological maximum of around 300 bpm [24]. This can be seen in Figure 4 where the heart rate peaks above 340 bpm briefly with a total average of 260 bpm, a relatively high value. The evaluation of these results necessitated the use motion artifact reduction. It should be noted that, to the best of our knowledge, there is no wireless, wearable and portable “gold standard” ECG system exist to be deployed on dogs in such outdoors field conditions to benchmark against since it is difficult to obtain ECG during such high intensity motion. Therefore, we used visual inspection of the data and the maximum heart rate normal values reported in the literature to assess the reliability of the outcomes. The authors are not aware of any commercially available products capable of performing ECG collection at maximal working speeds where an application specific wireless ECG system was custom designed with novel electrode structures enabling assessment of biopotentials despite the presence of hair and skin motion.

**Figure 5:**
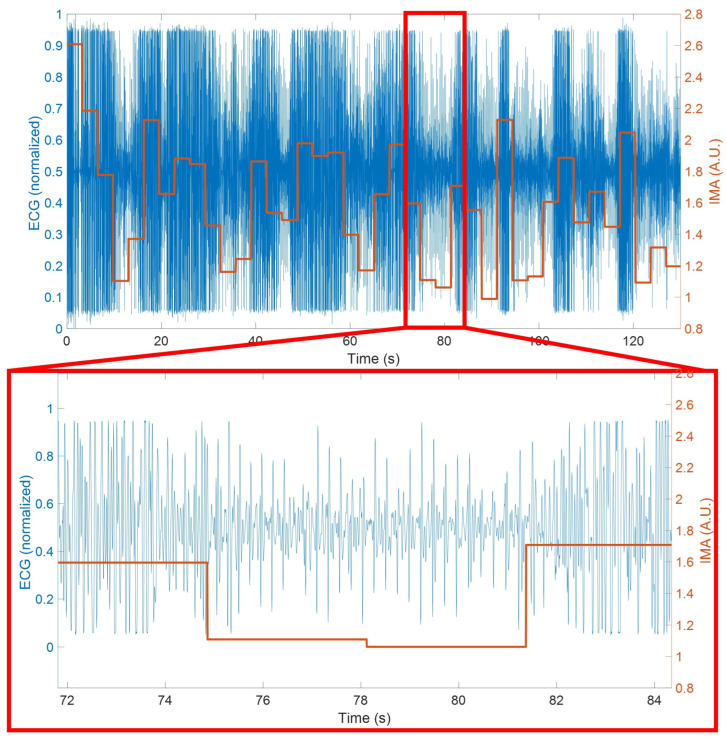
Process of heart rate estimation using adaptive filtering. The areas regions of ECG (blue) corresponding to lower IMA (orange) are used to estimate the overall heart rate and areas of higher IMA are removed.

**Figure 6:**
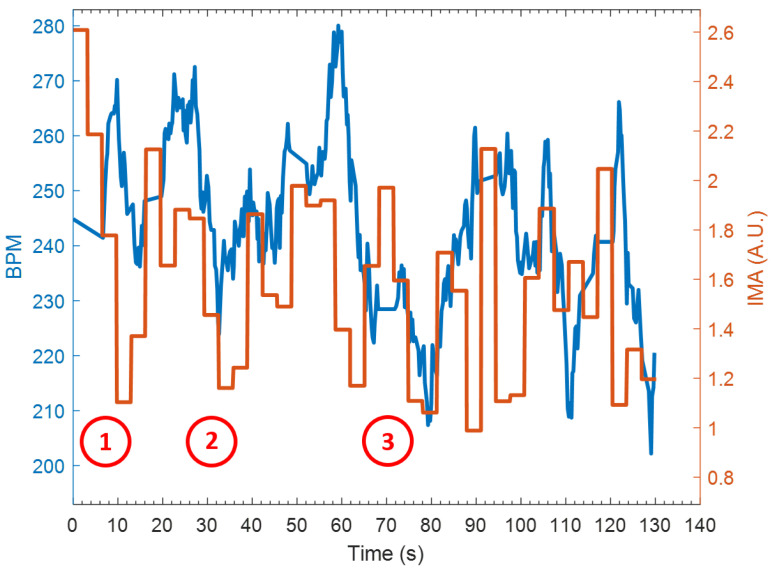
Heart rate estimation (blue) overlaid with the IMA (orange). The three bumper retrieval events are enumerated in red.

In order to improve heart rate estimation, the three axes of the IMU are vectorized:

$$\sqrt{a_{x}^{2}+a_{y}^{2}+a_{z}^{2}}$$

to produce a singular value. Then, the data are windowed into 5-s segments and the average acceleration vector is calculated per window. This affords a relative comparison across the experiment of whether or not the dog is increasing or decreasing activity for each segment. Finally, a total average is computed for each window and any epoch with a value above one standard deviation of the average is removed from the heart rate estimation as it contains excessive motion artifacts. As can be seen in Figure 5, when the vectorized value is below the aforementioned threshold, the signal quality greatly increases and the peaks become more observable visually. Utilizing the IMU for adaptive filtering by removing any activity one standard deviation above the average retains 84% of the data. This produces a more reasonable result for overall heart rate estimation shown in Figure 6. Once the highest activity data is removed, the average falls to 216 beats per minute with peaks around 280. This value is in line with [24] where heart rate was examined after extreme exercise.

While motion artifacts can be mostly addressed through ergonomics of electrode design and signal processing, the trend of the data collected indicates a potential additional improvement through careful design of the analog front end circuits by adding an adaptive gain stage in the future. The data collected in the field experiments suggest that the amplitude of the ECG signal is the highest at points of highest motion and as such, there’s a proportional relationship between the activity level and peak magnitude of the ECG signal. An adaptive amplifier could be utilized to perform amplification at an inverse relationship to the activity level, decreasing the gain when motion is high and increasing the gain during lower activity. This would improve the overall signal to noise ratio of the ECG signal.

In all, we ran four experiments with two different dogs performing two experiments each with a total of nine object retrievals. We examined the interplay between heart rate and activity level 10 s before and after for all nine retrievals as shown in Table 1. As a representative example in Figure 6, the three bumper retrievals are denoted by 1, 2, and 3 respectively. Overall, a similar trend is noted for the heart rate immediately before and after the retrievals. In seven of the nine retrievals, the heart rate tended to increase once the dog retrieved the object (located the bumper toy). Then, the dog would return to his handler and the heart rate recovered. This resulted in lower activity levels in six out of nine retrievals, and then both the heart rate and activity level would increase when the dog was released to continue its search.

While our aim here is to provide proof-of-concept data about the use of on-body and aerial sensors for tracking dogs, we note that our observations lead to interesting trends with a need for further investigation in the future, especially to explore the relationship between the increase in heart rate associated with the dog after object retrieval despite a decrease in activity level. The decrease in activity level generally occurred because the dog returned the bumper to the handler; however, it is interesting to note that in seven of the nine retrievals the heart rate starts to decrease as the bumper is retrieved and increases after retrieval. We keep this investigation beyond the scope of this article and reserve for a future study.

**Figure 7:**
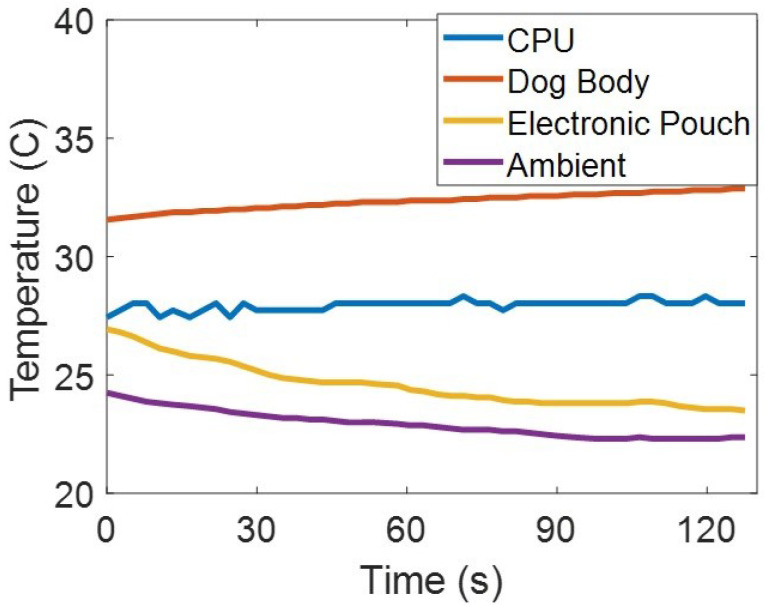
Measured temperatures from the electronics pouch, CPU, space between the dog body and the computer, and the ambient environment.

The temperature sensors distributed across various points on the harness also revealed interesting results. We collected the temperature inside the electronics pouch, the CPU temperature, the ambient temperature, and the temperature between the dog’s fur and the computer as shown in Figure 7. The temperatures were relatively constant across all measurement points with the largest change being a 3 °C drop in the electronic pouch temperature. The ambient temperature dropped approximately 2 °C, the CPU temperature remained relatively constant. We note the drop in ambient and electronic pouch temperatures could be explained by extra airflow over the sensor when the dog is moving at relatively high rates of speed. Allowing more airflow within the pouch mitigated the CPU temperature rises and subsequently CPU performance to decrease. Finally, the skin temperature of the dog’s under the harness increased by approximately 1 °C. As expected, the measured values were lower than the core body temperature for a healthy dog (approximately 38.5 °C measured rectally) due to the location of the sensor being over the skin and fur. It was in the expected range and still provides an endpoint to track overheating to prevent a potential heat stroke [35].

**Figure 8:**
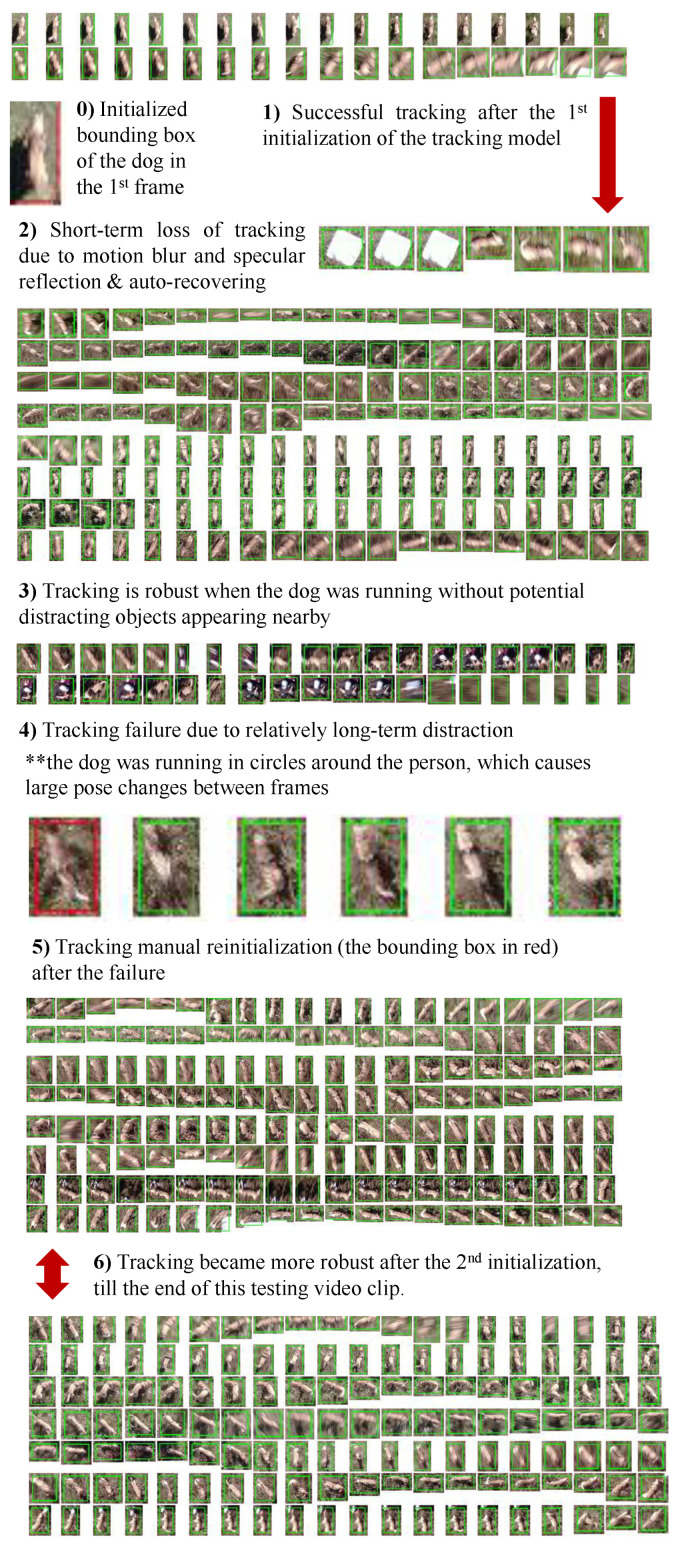
Examples of dog tracking results. We dissect the tracking results into seven components (0–6), illustrating the inner working mechanism of the tracking algorithm we used. Overall we observed that the tracking algorithm worked well on the videos we collected. We show the tracking bounding boxes every five frames. The bounding boxes in red represent either the initialized box (e.g., in the first frame) or re-initialized boxes (e.g., after the tracking failed due to the dog being completely occluded for a while from (4,5)). The bounding boxes in green are from online tracking. It also is observed that the tracking drifts due to fast motion caused either by the drone moving or by the dog running, e.g., in (2). Best viewed in magnification for checking the cropped individual tracking result.

**Figure 9:**
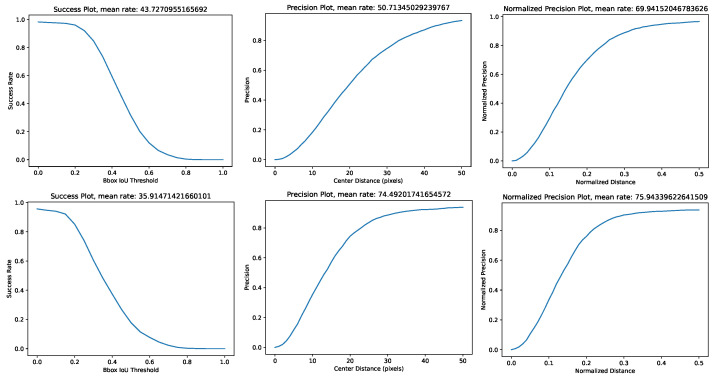
Quantitative results of dog tracking in two videos. The widely used success rate, precision and *normalized precision* are used in evaluation.

**Figure 10:**
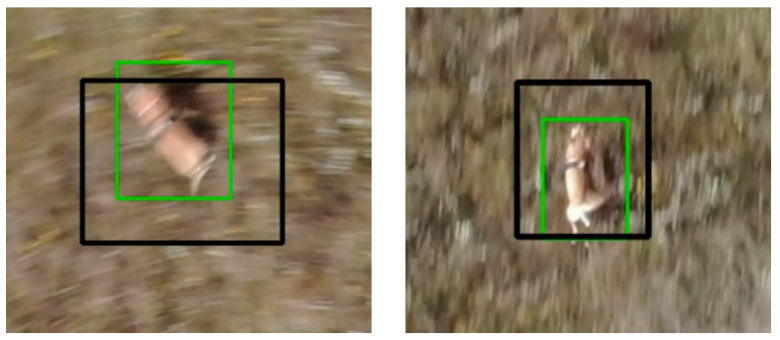
Illustration of the lack of compactness of the annotated bounding boxes (in black) in some frames, which may cause the not-very-high success rate in evaluation. The bounding boxes in green are tracking results.

Figure 8 presents representative examples of dog tracking results using UAV camera images and Figure 9 demonstrates that a satisfactory success rate, precision and *normalized precision* performance were achieved in the scope of this study. Two other representative image examples are provided in Figure 10 illustrating the lack of compactness of the annotated bounding boxes in some frames affecting the success rate evaluation in Figure 9. The next and future step would be the development of a fully automatic dog tracking algorithm to eliminate the multi-frame initialization entailed in the current experiments (e.g., two initialization frames are used in (0) and (5) in Figure 8). To eliminate the manual initialization, one potential solution is to integrate object detection and online object tracking. The former can provide high-scoring detection results (e.g., dogs) to initialize the latter, and also help correct the drift of tracking.

## 4. Discussion

In this paper, we present the first study fusing on-body wearable sensors and aerial-visual sensors to monitor working dogs. We particularly focus on offering solutions towards assessment of two important signals while the dog is performing a high speed scent-detection task in a large area. These signals include the heart rate acquired with the on-body ECG electrodes and macro-level activity of the dog using visual sensors. In this section, we would like to widen the scope and offer a broader vision that can be achieved with such stream of dataflow acquired from a suite of multimodal on-body and aerial sensors in real time. This novel data stream could be essential to determine the true performance of the working dog, characterize confidence in searches, improve situational awareness, and optimize search patterns to increase effectiveness while also ensuring welfare of working dogs.

Due to space limitation, several aerial-sensor enabled macro-environment analysis is kept beyond the scope of this paper, including UAV cameras examining the topology of the land to identify certain characteristics that would impact search performance (e.g., terrain that may impact airflow; moisture that may impact scenting conditions; or potential hazards on the search trajectory). The fusion of aerial UAV cameras and on-body IMUs and global positioning systems (GPS) could offer very high resolution tracking of the dog throughout the search whether it is on- or off-sight of the visual sensors. Beyond providing a live video feed of the surveyed area, the UAVs can also be used as wireless data relays to transmit any information received from electronics carried by humans or dogs to distant base stations or directly to the Cloud [8,9]. The typical quadcopter UAVs are capable of approximately 30 min of flight time and fixed wing devices can fly longer. The extension of flight duration in UAVs is beyond the scope of this paper and an active research under the field of aerospace engineering. The presented effort on drone-wearables connection will definitely benefit from those advancements in the long run. In order to perform an extensive search and rescue, multiple drones and a mesh network would be required. A protocol would need to be developed to hand-off the data transmission to ensure continuous monitoring. Additionally, the dogs should be trained to perform the task with an operating drone present. This would ensure the dogs are able to communicate and receive commands if necessary or learn to ignore the drone and focus on the scent detection task [16,25].

The combined on-body and aerial sensing of the working dog and the environment could also comprise a basis for novel in vivo psycho-physiological studies of working canine behavior as well as avenues for advanced algorithmic analysis and optimization of handler-canine-drone triad performance in real-world settings. Working canines are among the most sophisticated mobile olfactory sensors in existence, and it is well documented that air conditions (flow, moisture, temperature, wind direction, ground influences, etc.) heavily influence the performance of these working animals. For example, the way air swirls up and over an obstacle can lead to cyclic scent flows emanating from the obstacle. Alternatively, on a warm, high-humidity day a dog may generate excessive body heat in low-lying terrain where airflow is stalled by vegetation, thereby reducing the dog’s olfactory effectiveness. The tracking of micro- and macro-level factors with environmental sensors placed on harness and aerial visual sensors (such as hyperspectral imaging) could provide a better assessment of the dogs working conditions and effect of these on the its success rate for scent detection tasks. Moreover, combination these with tracking of the heart rate, respiratory patterns and movement of the body could further improve this success rate [9,15,17].

The system presented in this study with the addition of other commercially available sensors and more advanced data analytics hold promise for such a grand vision that will be the focus of our future work on this topic.

## 5. Conclusions

In this paper, we used on-body and aerial sensing to monitor the behavior, physiology, and environment of a dog during search and rescue tasks. We successfully measured heart rate, activity level, and temperature via on-body sensors placed on a wearable harness. We used commercial UAVs with cameras for aerial tracking of the dog throughout the experiment. Taken together, these two categories of capability substantively increase the ability to monitor and characterize the performance of working dogs in vivo. Wearable and drone-based sensing combined enable simultaneous collection of both *egocentric* and *allocentric* data representing canine performance and welfare, as well as location within the environment and interaction with specific objects. This capability goes beyond simply using GPS for geolocation to enable a system whereby canine behavior can be analyzed in concert with environmental factors discernible by drone-based environmental sensing.

We particularly focused on design enhancements to address experimental challenges imposed by high intensity motion activity of dogs performing scent detection tasks in uncontrolled environments. We achieved a robust wireless network encompassing a nearly two acre space and allowing for real-time data streaming. In this space, our proof of concept experiments included two dogs and nine search-and-retrieval sessions. It is very challenging to acquire ECG signals during excessive motion of working dogs. We improved and tested the reliability and robustness of an application-specific 3D printed electrode during maximal working speeds. A custom designed leather harness improved the ergonomic fit and proved rugged enough to withstand the uncontrolled, outdoor environment. The heart rate estimation was improved through adaptive filtering by using the activity level from the IMU to remove excessive motion during the bumper retrieval. Signal processing techniques recovered 84% of the data in an application where it was not possible to collect usable data previously. In general, we saw the dog’s heart rate increase upon retrieval of the bumper and recover after the retrieval was complete and it waited to be released.

Using deep neural networks applied to UAV visual recordings for object tracking, we accurately tracked dogs’ movements throughout a two acre field. With this, we performed automated video tracking of dogs in the wild and obtained promising results. We analyzed the barriers towards improving the tracking performance further and offered suggestions for a fully automatic tracking algorithm by integrating DNN object detection and online object tracking in the future.

While many challenges exist to realize the full capabilities of automated canine-human-drone triads, the work presented in this paper represents a significant effort forward in overcoming some of the most significant ones. Fusing data streams obtained from on-body and aerial sensors could enable more efficient performance of remote scent detection search tasks and ensure the welfare of the working dog during potentially dangerous environments.

## Figures and Tables

**Table 1:** Heart rate and activity level trends ten seconds before and after bumper retrieval.

	Heart Rate			Activity Level		
	**Before**	**After**	**Trend**	**Before**	**After**	**Trend**
Dog 1	240	270	Increase	1.94	1.31	Decrease
Dog 1	270	270	No Change	1.57	1.51	Decrease
Dog 1	228	240	Increase	1.82	1.70	Decrease
Dog 1	192	234	Increase	2.00	1.85	Decrease
Dog 1	252	252	No Change	1.92	1.71	Decrease
Dog 1	258	282	Increase	1.30	1.63	Increase
Dog 2	234	264	Increase	2.19	1.53	Decrease
Dog 2	282	288	Increase	1.50	1.54	Increase
Dog 2	248	266	Increase	1.35	1.39	Increase

## Data Availability

Data available upon request.
